# Hypertriglyceridemia-induced acute pancreatitis in children: A mini-review

**DOI:** 10.3389/fped.2022.931336

**Published:** 2022-08-25

**Authors:** John M. Grisham, Andrew H. Tran, Kate Ellery

**Affiliations:** ^1^Division of Gastroenterology, Hepatology, and Nutrition, Nationwide Children's Hospital, Columbus, OH, United States; ^2^The Heart Center, Nationwide Children's Hospital, Columbus, OH, United States; ^3^Department of Pediatrics, The Ohio State University, Columbus, OH, United States; ^4^Division of Gastroenterology, Hepatology, and Nutrition, UPMC Children's Hospital of Pittsburgh, Pittsburgh, PA, United States

**Keywords:** hypertriglyceridemia, pancreatitis, children, pediatric, triglyceride

## Abstract

Severe hypertriglyceridemia (HTG) is a known metabolic cause of acute pancreatitis (AP) in pediatric patients. The incidence of hypertriglyceridemia-induced acute pancreatitis (HTG-AP) is less well established in pediatric compared to adult patients. Studies in adults suggest that higher risk of AP occurs when triglyceride levels (TG) are >1,000 mg/dL. Most common etiologies for severe HTG in pediatric patients are either from primary hypertriglyceridemia, underlying genetic disorders of lipid and TG metabolism, or secondary hypertriglyceridemia, separate disease or exposure which affects TG metabolism. Most common theories for the pathophysiology of HTG-AP include hydrolysis of TG by pancreatic lipase to free fatty acids leading to endothelial and acinar cell damage and ischemia, as well as hyperviscosity related to increased chylomicrons. Though there are varying reports of HTG-AP severity compared to other causes of AP, a steadily growing body of evidence suggests that HTG-AP can be associated with more severe course and complications. Therapeutic interventions for HTG-AP typically involve inpatient management with dietary restriction, intravenous fluids, and insulin; select patients may require plasmapheresis. Long term interventions generally include dietary modification, weight management, control of secondary causes, and/or antihyperlipidemic medications. Though some therapeutic approaches and algorithms exist for adult patients, evidence-based management guidelines have not been well established for pediatric patients.

## Introduction

Hypertriglyceridemia-induced acute pancreatitis (HTG-AP) is a recognized but incompletely characterized disease in children and adolescents. Early accounts of an association between severe hypertriglyceridemia (HTG) and pancreatitis date back to the mid-1800's (1). In adults, HTG-AP is one of the most common identified causes of acute pancreatitis (AP) after gallstones and alcohol, accounting for an estimated 1–10% of cases (2–6). The overall annual incidence of AP in children is estimated between 3.6–13.2/100,000 persons per year and increasing, potentially related to increased awareness/testing vs. true increased incidence (7, 8); however, some reports have found annual incidence of Pediatric AP may be stabilizing (9). Idiopathic AP accounts for as many as 13–37% of pediatric cases, with identified causes including biliary disease 10–30%, medications <25%, and variable incidence of infection, trauma, systemic diseases, metabolic diseases (including HTG-AP), and hereditary causes (10, 11). The incidence of HTG-AP in children is not well quantified, but an estimated 2–7% of AP is secondary to HTG or the category “metabolic causes” (10–13). Published data on pediatric HTG-AP is limited. The purpose of this review is to describe the etiologies, clinical features, acute management, and prevention of HTG-AP and to highlight existing literature and clinical guidelines targeting HTG-AP in pediatric patients.

## Etiologies and pathophysiology of hypertriglyceridemia

### Primary hypertriglyceridemia

Primary Hypertriglyceridemias typically caused by monogenic or multifactorial defects resulting in dysfunctional triglyceride (TG) synthesis or metabolism. Some of the most common disorders are summarized below (14, 15).

#### Chylomicronemias

Causes of severe HTG can be divided into monogenic chylomicronemia and multifactorial/ polygenic chylomicronemia.

Monogenic chylomicronemia is an autosomal recessive condition also known as familial chylomicronemia syndrome. Mutations in one of five genes, *LPL, APOC2, APOA5, LMF1, GPIHBP1* result in deficiency of lipoprotein lipase (LPL), Apolipoprotein C-II, Apolipoprotein A-V, lipase maturation factor 1, or GPIHBP1, respectively (15). In this condition, chylomicron accumulation results in high fasting TG levels and reduced High-Density Lipoprotein (HDL) and Low-Density Lipoprotein (LDL) (16). Recurrent pancreatitis is common (17) due to severe HTG.

As indicated by its name, multifactorial/polygenic chylomicronemia can be caused by multiple factors including heterozygous variants in the aforementioned monogenic chylomicronemia genes or from combination of several TG-raising polymorphisms leading to clinical manifestations similar to monogenic chylomicronemia (15).

#### Other causes of primary HTG

Causes of less severe HTG can be categorized into three groups: multifactorial/polygenic HTG, combined hyperlipoproteinemia, and dysbetalipoproteinemia.

Multifactorial/polygenic HTG, previously known as familial hypertriglyceridemia, has no currently identified genetic locus. HTG typically manifests in adulthood; however, pediatric expression has increased due to childhood obesity (18–20). This condition results in Very Low-Density Lipoproteins (VLDL) overproduction and impaired catabolism of TG-rich lipoproteins resulting in HTG (14, 21). Typically, patients are asymptomatic with HTG between 250 and 1,000 mg/dL (22).

Combined hyperlipoproteinemia, previously named familial combined hyperlipidemia, has multiple genetic loci and complex pathophysiology with variable expressivity (23, 24). Combined hyperlipoproteinemia is typically characterized by overproduction of VLDL and apolipoprotein B-100, reduction in fatty acid uptake by adipocytes, and decreased clearance of chylomicron remnants (14). These patients may exhibit LDL elevation in addition to HTG (15).

Dysbetalipoproteinemia is caused by a combination of polygenic contributors in addition to apolipoprotein-E mutation (15). This results in abnormal metabolism of chylomicrons, Intermediate Density Lipoprotein, and VLDL remnant particles leading to elevated total cholesterol and TG (25). It is not typically expressed in childhood unless there is secondary exogenous risk (26, 27).

### Secondary hypertriglyceridemia

Secondary hypertriglyceridemia results from many diseases, exposures, and underlying risk factors. Blackett et al. report genetic background and developmental factors play a significant role in the risk for secondary HTG (28). Heterozygous relatives of patients with primary dyslipidemias can develop severe dyslipidemia/HTG, further worsened by other factors such as alcohol, obesity, and high-risk medications (29, 30). Features of growth and development such as intrauterine growth restriction, prematurity, childhood obesity, and puberty also increase risks for dyslipidemia and HTG (28).

Type 1 Diabetes and insulin deficiency, at baseline and in extremis such as Diabetic Ketoacidosis (DKA), have known associations with elevated TG and cholesterol which improve with insulin therapy (31–33). Conversely, insulin resistance, in obesity and/or Type 2 Diabetes, can lead to increased serum free fatty acids (FFA) and insulin-stimulated hepatic TG synthesis which increases VLDL and TG levels (34). Additionally, chylomicron production (and resulting HTG) is less susceptible to insulin suppression in insulin resistant patients (35).

Pediatric disorders in other organ systems that have association with HTG include diseases of liver (non-alcoholic fatty liver disease, hepatitis C, type 1 glycogen storage disorder), kidney (nephrosis), endocrine (hypothyroidism, growth hormone deficiency/excess, congenital generalized lipodystrophy), and immune system (human immunodeficiency virus (HIV) lipodystrophy, gammopathies) (28). Additionally, many medications have known and some unknown mechanisms that lead to HTG including glucocorticoids, L-asparaginase, oral estrogens, retinoids, immune suppressants, protease inhibitors, bile acid sequestrants, loop/thiazide diuretics, beta-blockers, and alcohol (28).

### Pathophysiology of hypertriglyceridemia-induced acute pancreatitis

The pathophysiology of HTG-AP is not well characterized. One theory proposed by Havel et al. suggests that pancreatic lipase hydrolyzes excess TG in pancreatic capillary beds leading to high concentration of FFAs; these FFAs aggregate causing damage to acinar and capillary endothelial cells with resulting ischemia, increased acidity, and further FFA toxicity (36, 37); additionally, chylomicrons may increase serum viscosity, further decreasing pancreatic blood flow and adding to ischemic/acidotic environment (36–38).

## Clinical definitions and presentation

Diagnosis of pediatric AP requires meeting ≥2 of 3 criteria including: (1) abdominal pain compatible with acute pancreatitis, (2) serum lipase and/or amylase level ≥3 times upper limit of normal, (3) imaging findings consistent with acute pancreatitis (39). HTG-AP frequently presents similarly to other causes of AP; however, certain features in patient history (obesity, alcohol use, diabetes), family history (hyperlipidemia, early cardiac death), physical exam (eruptive or tuberous xanthomas, lipemia retinalis, hepatosplenomegaly), and laboratory evaluation (lipemic or “milky” appearing serum) may raise suspicion for hypertriglyceridemia (2, 40, 41).

Triglyceride levels in pediatric patients are considered “high” at the 95th percentile for age, specifically, TG >100 mg/dL (0–9 years old) or >130 mg/dL (10–19 years old) (42). While fasting TG levels >200–499 mg/dL are defined as higher risk and recommendations are made to consider pharmacotherapy, no further stratification is defined for TG levels >500 mg/dL. Shah et al. combined the Endocrine Society values for adult severe HTG and the Pediatric Expert Panel recommendations to better delineate classification and risk for children with TG ≥500 mg/dL with additional categories for “Very High” (≥ 500-999 mg/dL), “Severe” (≥1,000–1,999), and “Very Severe” (≥2,000 mg/dL) (14, 43). The threshold at which HTG can cause AP is debated. Commonly cited levels are between 1,000–1,772 mg/dL (41, 44) with some reports as low as 500–1,000 mg/dL (45). The risk of AP with TG levels <1,000 mg/dL is not well defined, however, the lifetime risk of AP in severe HTG >1,000 mg/dL has been estimated at ~5 and 10–20% for very severe HTG >2,000 mg/dL (46).

There are varying reports on the clinical course, severity, and complications in HTG-AP compared to other causes of AP. HTG has been shown to affect severity of AP in animal models (47, 48). *Ex-vivo* studies have demonstrated that triglycerides may play a role in AP-associated respiratory failure (49). Some reports did not find a difference in morbidity and mortality between HTG-AP and other causes of AP (2, 50); however, the threshold for HTG in at least one prospective study was >175 mg/dL, which is lower than typically seen in HTG-AP. Conversely, there have been several studies which demonstrated increase in severity, recurrence, hospital stay, Intensive Care Unit (ICU) care, incidence of pancreatic necrosis, abscess formation/other infection, and renal failure in patients with HTG-AP compared to other causes of AP (51–54). Despite mounting evidence of increased severity in HTG-AP, actual TG level likely does not directly correlate with severity (50, 53).

## Treatment of hypertriglyceridemia-induced acute pancreatitis

The treatment goals of HTG-AP are to lower TG levels and prevent recurrence of AP. Patients with HTG >1,000 mg/dL plus AP or abdominal pain (symptomatic HTG) typically require hospital admission for aggressive interventions to minimize the risk of complications (55, 56). For asymptomatic severe HTG (>1,000 mg/dL) without confirmed AP, reasons for hospitalization include uncontrolled diabetes, HTG at a level where AP previously occurred, continued exposure to trigger that can increase TG levels, or pregnancy in third trimester.

### Initial management of acute episode

#### Intravenous fluids and diet

Fluid management recommendations include initial 10–20 ml/kg boluses of lactated ringers or normal saline fluids based on hydration/hemodynamic status followed by continuous intravenous fluids (IVF) at 1.5–2x maintenance rate (57). Current recommendations in both adult and pediatric literature suggest that high-rate IVF and early enteral nutrition (unless contraindicated or not feasible) decrease length of hospital stay and risk of mortality for acute pancreatitis patients (57, 58). In contrast, the first step of therapy for HTG-AP involves dietary restriction/*nil per os* (NPO). Limiting enteral nutrition can decrease production of diet-derived chylomicrons. This also facilitates clearance of already present chylomicrons and reduces TG (59). Once TG levels are <500 mg/dL, patients can gradually increase fat intake to a goal 10–15% of total dietary calories while monitoring TG levels (28, 41, 43).

#### Insulin

Insulin can increase activation of LPL which increases clearance of chylomicrons and decreases levels of TG (31). Insulin is effective for both HTG and hyperglycemia in diabetic patients (60, 61) but also can be used to treat HTG in non-diabetic patients (62, 63). Euglycemia should be maintained with dextrose fluids in non-diabetic patients. Both intravenous (IV) and subcutaneous dosing have been used successfully (62, 63), but continuous IV Insulin has the benefit of easier titration; though no society guidelines for continuous insulin dosing in HTG patients were found, Schaefer et al. have suggested continuous insulin drip 0.1–0.3 U/kg/hour with dextrose fluids to maintain blood glucose between 140– 80 mg/dL (55). Insulin can reduce TG level up to 40% in the first 24 h (60) and between 50–75% over 2–3 days (64); even further reduction up to 80% in the first 24 h is possible when kept NPO (61). One small (*n* = 17) retrospective pediatric HTG-AP cohort study from Ippisch et al. showed statistically significant difference (*P* = 0.0339) in mean 24-h reduction of TG by 40% with insulin vs. 17% without insulin (65).

#### Plasmapheresis

In adult HTG-AP, plasmapheresis can effectively reduce TG levels rapidly by 40–70% after a single treatment (40, 66, 67). Multiple case reports demonstrate the utility of plasmapheresis in patients with concomitant severe disease such as DKA or complications of HTG-AP such as acute respiratory distress syndrome (66, 68). The main indications for plasmapheresis include severe HTG-AP with worsening organ dysfunction/multi-organ failure, worsening systemic inflammation, or lactic acidosis (69, 70). Evidence of improvement in clinical outcomes from plasmapheresis varies. Chen et al. (71) did not show a statistical difference in morbidity or mortality between plasmapheresis vs. no plasmapheresis groups, though this was partially attributed to delay in initiation. Plasmapheresis for HTG-AP has relatively fewer published reports in pediatric patients (68, 72). One limitation in pediatric patients is the availability of equipment, protocols, and providers to effectively manage therapeutic plasmapheresis for HTG reduction. If the patient cannot tolerate plasmapheresis or it is not available, providers should strongly consider other interventions, such as continuous insulin even in non-diabetic patients (40).

#### Heparin

Heparin stimulates LPL release *in vivo* (31) from several extrahepatic tissues such as myocytes, adipose tissues, and macrophages; however, after initial peak in LPL, serum levels rapidly drop likely due to uptake and degradation in the liver (73). Additionally, prolonged use can deplete LPL stores, allowing rebound in TG levels. There has also been some reluctance to use heparin in cases of pancreatic necrosis due to risk of hemorrhage (74). The routine use of heparin in the management of HTG-AP might be limited due to the above features.

### After stabilization

**Figure 1:**
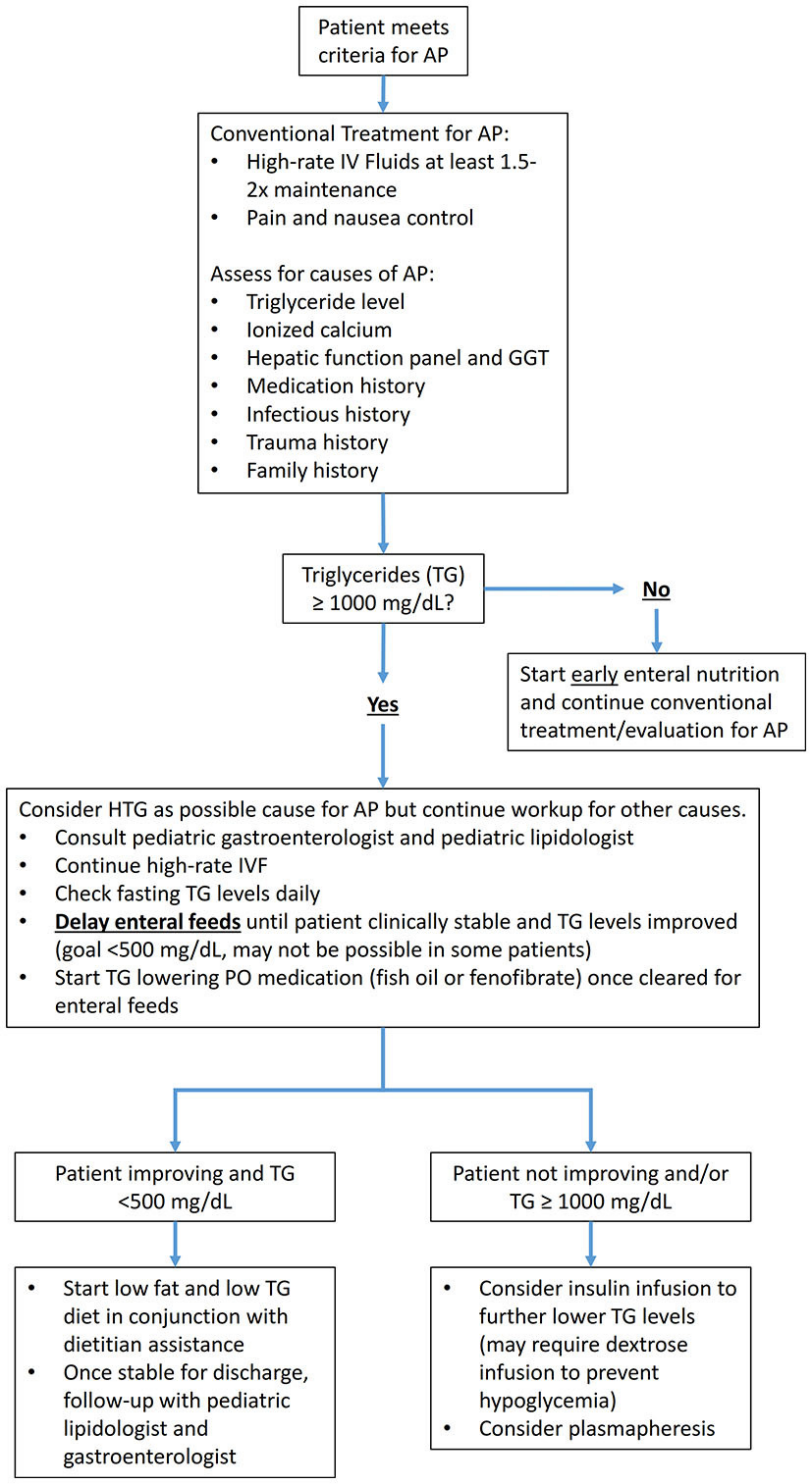
Algorithm for management of HTG-AP in children.

For acute HTG-AP, consensus TG treatment goal varies; this may be related to the relative risk at >500 mg/dL (40, 46, 65, 69) vs. absolute risk at >1,000 mg/dL (43, 55, 56) for development of AP and other complications. The decision on when to discontinue higher level interventions and advance diet should be determined based on individual patient factors and feasibility of attaining goal TG level. Certain factors such as excessive post-prandial TG rise in genetic hypertriglyceridemia may warrant goal <500 mg/dL (56), but further evidence for this recommendation is needed. All patients should be counseled on the need for long term interventions including dietary fat restriction, weight management, and exercise. Patients at risk for persistent HTG should be started on oral antihyperlipidemic agents during hospitalization. An algorithm (Figure 1) summarizing pertinent management steps for Acute HTG-AP has been created by adapting various external references (40, 55, 65, 69).

## Long-term management/prevention

### Screening

The American Academy of Pediatrics (AAP) recommends universal lipid screening for all children between 9–11 and 17–21 years old. Additional screening for dyslipidemia should be considered in any patient 2 years or older with any one of the following: (1) parent, grandparent, aunt/uncle, or sibling with history of a heart attack, angina, stroke, coronary artery bypass/stent/angioplasty, or sudden death in males <55 years and females <65 years. (2) Parent with a total cholesterol ≥240 mg/dL or known dyslipidemia. (3) Patient has diabetes, hypertension, BMI >85th percentile, or smokes cigarettes. (4) Patient has other moderate- or high-risk medical conditions including chronic kidney disease/post-renal transplant, post-orthotopic heart transplant, Kawasaki disease with current or regressed coronary aneurysms, chronic inflammatory disease, HIV, and nephrotic syndrome (42).

### Dietary and lifestyle interventions

Management of HTG in the outpatient setting is primarily driven by lifestyle and dietary changes. Per the CHILD-2 diet from the AAP, dietary management of HTG includes reducing the amount of daily calories from fat to 25–30% with ≤7% from saturated fat, limit monounsaturated fat to ~10%, limit cholesterol intake to <200 mg/d, avoid trans fats, reduce simple carbohydrate such as sugar-sweetened beverages, and increasing dietary fish to raise omega-3 fatty acid intake (42). For severe HTG, daily fat intake should be further decreased to 10–15% of total calories (41, 75).

Attention should be paid to prevent deficiency of essential fatty acids, linoleic acid (LA) and α-linolenic acid (ALA), as well as fat soluble vitamins (28). The minimum recommended intake to prevent essential fatty acid deficiency is ≥10% of total calories from polyunsaturated fats (76) with 2–4% of calories/energy from LA and 0.25–0.5% from ALA (77, 78); however, it is advisable to consult with a clinical dietician to ensure that each patient has their own individualized nutrition plan. Other interventions to manage HTG include increasing physical activity with the most recent guidelines recommending >60 minutes of moderate-to-vigorous physical activity daily for children aged 6–17 years (79–81).

### Pharmacotherapy

Several drugs are available to lower TG when diet and lifestyle interventions are insufficient (Table 1). Fibrates and omega-3 fatty acids are the two most common therapies used to treat HTG particularly in the outpatient setting (82, 83). While fibrates are not FDA approved for use in children, they are generally tolerated well and are considered part of the armamentarium in managing significant HTG in children (83).

**Table 1:** Summary of pharmacotherapy options for HTG-AP in children.

**Medication**	**Indications**	**Effects**	**Comments**
Fibrates	Maintenance/Preventive	TG ↓↓ LDL-C ↓ (mild) HDL-C ↑ (mild)	Used off-label in children; monitor for hepatic and muscle side effects.
Omega 3 fatty acids	Maintenance/Preventive	TG ↓ LDL-C - variable ↑/↓ HDL-C - variable ↑/↓	Goal is ~4 g/day of EPA +DHA; no hepatic or muscle side effects; can be used in combination with statins or fibrates
Statins	Maintenance/Preventive	TG ↓ (mild) LDL-C ↓↓ HDL-C ↑	Not used primarily for TG lowering but can be used if patient has combined TG and LDL-C elevation
Insulin	Acute Severe HTG	TG ↓↓ Glucose ↓↓	See acute treatment section
Heparin	Acute Severe HTG	TG ↓↓ (risk to ↑)	See acute treatment section

In adults, fibrates can lower TG levels by 46–62% with isolated hypertriglyceridemia and 24–36% in mixed dyslipidemia (84). There is limited safety data in pediatric patients regarding long term treatment with fibrates both alone and in combination with statins (85, 86). Nevertheless, a review of National Health and Nutrition Examination Survey data from 1999–2006 found that fibrates were the most commonly prescribed TG lowering medication in children with HTG >500 md/dL (87). Additionally, Manlhiot et al. described a statistically significant decrease in TG levels using fibrate therapy in children, though there was no specification of agent or dose (20). One drawback with fibrates is length of time from initiation to clinical effect (63).

Omega-3 fatty acids are frequently used as adjunctive agents for TG management. Adult studies have shown a mean reduction of ~45% with 4g per day dosing (88). However, some pediatric studies have not shown significant change with either low dose (500–1,000 mg daily) or high dose (3,360 mg daily) of Omega-3 fatty acids (89, 90).

Niacin (Nicotinic Acid) is another medication used in adult patients for TG/cholesterol control. One of the use-limiting adverse effects of niacin is Prostaglandin E2 mediated flushing, which can be improved by taking aspirin prior (84), though aspirin can lead to Reye syndrome in younger children. A study by Colletti et al. showed niacin was effective at reducing total and LDL cholesterol in children but did not lower triglycerides; additionally, reversible adverse effects were seen in 76% of study participants and discontinuation of niacin due to adverse effects occurred in 38% of patients (91). Niacin is no longer routinely recommended for treatment of dyslipidemia due to this side effect profile.

Statins are widely prescribed antihyperlipidemic agents that do have well known utility in pediatric patients (87) and have approval from the Food and Drug Administration for 8–18 year old patients for treating elevated LDL-C or non-HDL-C with HTG and other risk factors (42). However, the efficacy of TG lowering effects from statins can vary (20, 92).

## Conclusion

Hypertriglyceridemia is a known metabolic cause of acute pancreatitis in adults and increasingly recognized in children. HTG can be associated with primary/genetic causes or secondary causes (insulin dysregulation, medications, and other systemic diseases). TG levels > 1,000 mg/dL are most commonly associated with risk for developing acute pancreatitis, though there may be risk if > 500 mg/dL. Presentation for HTG-AP is similar to other causes of AP, but features such as obesity, diabetes, pregnancy, alcohol/high risk medication use, familial dyslipidemia, or exam findings of HTG may suggest the diagnosis and outcomes may be more severe. Several acute interventions (dietary restriction, insulin, plasmapheresis, heparin) and preventative measures (limiting dietary fat, exercise/weight loss, oral antihyperlipidemic medications) are available, but most outcome data on these interventions and management algorithms are focused on adult patients. For example, early plasmapheresis vs. reserving invasive interventions for severe disease/lack of improvement is currently contested in adult HTG-AP patients, but lower relative availability and evidence-based outcomes for plasmapheresis in pediatric patients presents a notable barrier. Further studies are needed to refine the therapeutic approach to pediatric HTG-AP.

## Author contributions

JG, KE, and AT contributed to the conception of review and structure. JG performed the literature review and wrote the first draft of the manuscript. KE and AT contributed additional literature sources for review and content revision. All authors contributed to manuscript revision and approved of submitted version.

## Conflict of interest

The authors declare that the research was conducted in the absence of any commercial or financial relationships that could be construed as a potential conflict of interest.

## Publisher's note

All claims expressed in this article are solely those of the authors and do not necessarily represent those of their affiliated organizations, or those of the publisher, the editors and the reviewers. Any product that may be evaluated in this article, or claim that may be made by its manufacturer, is not guaranteed or endorsed by the publisher.
